# Correlates of facility delivery for rural HIV-positive pregnant women enrolled in the MoMent Nigeria prospective cohort study

**DOI:** 10.1186/s12884-017-1417-2

**Published:** 2017-07-14

**Authors:** Nadia A. Sam-Agudu, Christopher Isah, Chinenye Fan-Osuala, Salome Erekaha, Habib O. Ramadhani, Udochisom Anaba, Olusegun A. Adeyemi, Grace Manji-Obadiah, Daniel Lee, Llewellyn J. Cornelius, Manhattan Charurat

**Affiliations:** 1 International Research Center of Excellence, Institute of Human Virology Nigeria, Plot 252 Herbert McCaulay Way, Abuja, Nigeria; 2 Division of Epidemiology and Prevention, Institute of Human Virology, University of Maryland School of Medicine, Baltimore, USA; 3 Clinical Department, Institute of Human Virology Nigeria, Abuja, Nigeria; 4 University of Maryland School of Medicine, Baltimore, USA; 5 School of Social Work and College of Public Health, University of Georgia Athens, Athens, USA

**Keywords:** Pregnancy, HIV, Vertical transmission, Mentor mothers, Rural populations, Nigeria

## Abstract

**Background:**

Low rates of maternal healthcare service utilization, including facility delivery, may impede progress in the prevention of mother-to-child transmission of HIV (PMTCT) and in reducing maternal and infant mortality. The MoMent (Mother Mentor) study investigated the impact of structured peer support on early infant diagnosis presentation and postpartum maternal retention in PMTCT care in rural Nigeria. This paper describes baseline characteristics and correlates of facility delivery among MoMent study participants.

**Methods:**

HIV-positive pregnant women were recruited at 20 rural Primary Healthcare Centers matched by antenatal care clinic volume, client HIV prevalence, and PMTCT service staffing. Baseline and delivery data were collected by participant interviews and medical record abstraction. Multivariate logistic regression with generalized estimating equation analysis was used to evaluate for correlates of facility delivery including exposure to structured (closely supervised Mentor Mother, intervention) vs unstructured (routine, control) peer support.

**Results:**

Of 497 women enrolled, 352 (71%) were between 21 and 30 years old, 319 (64%) were Christian, 245 (49%) had received secondary or higher education, 402 (81%) were multigravidae and 299 (60%) newly HIV-diagnosed. Delivery data was available for 445 (90%) participants, and 276 (62%) of these women delivered at a health facility. Facility delivery did not differ by type of peer support; however, it was positively associated with secondary or greater education (aOR 1.9, CI 1.1–3.2) and Christian affiliation (OR 1.4, CI 1.0–2.0) and negatively associated with primigravidity (OR 0.5; 0.3–0.9) and new HIV diagnosis (OR 0.6, CI 0.4–0.9).

**Conclusions:**

Primary-level or lesser-educated HIV-infected pregnant women and those newly-diagnosed and primigravid should be prioritized for interventions to improve facility delivery rates and ultimately, healthy outcomes. Incremental gains in facility delivery from structured peer support appear limited, however the impact of duration of pre-delivery support needs further investigation. Religious influences on facility delivery and on general maternal healthcare service utilization need to be further explored.

**Trial Registration:**

ClinicalTrials.gov number NCT01936753, registered September 2013.

**Electronic supplementary material:**

The online version of this article (doi:10.1186/s12884-017-1417-2) contains supplementary material, which is available to authorized users.

## Background

Nigeria has significant gaps in its prevention of mother-to-child transmission of HIV (PMTCT) program performance. Only 30% of HIV-positive Nigerian women receive antiretroviral drugs (ARVs) for PMTCT, and only 12% of pregnant HIV-positive women delivering receive anti-retroviral therapy (ART) [1, 2]. Nigeria has the highest incidence and prevalence of child HIV globally, with an estimated 41,000 children under 15 years newly infected with HIV in 2015 [3]. New child HIV infections in Nigeria declined by only 21% between 2009 to 2015, the lowest decline amongst all Global Plan countries [2]. In addition, at 13.1% and 23.0% respectively, Nigeria has the highest six-week and final mother-to-child transmission rates globally [2]. Finally, at 9%, Early Infant Diagnosis (EID) uptake in Nigeria is unacceptably low [2].

Access to facility-based services is important for optimal PMTCT outcomes. Women who deliver outside health facilities are less likely to receive ARVs and quality obstetric care, and may suffer complications leading to vertical HIV transmission and maternal and infant mortality [4–6]. These risks are especially high in rural areas, where health service utilization is low [7, 8]. Only 47% of women in rural Nigeria access skilled antenatal care (ANC), compared to 86% of urban women [7], and only 22% of rural women deliver at health facilities, compared to 62% of urban women [7]. Furthermore, facility delivery data is available for <25% of HIV-positive pregnant Nigerian women [9]. Clearly, interventions to improve health-seeking behavior among rural women will be important for PMTCT in Nigeria.

The MoMent (Mother Mentor) Nigeria study evaluated the impact of structured peer support on EID uptake and maternal PMTCT retention [10]. Mentor Mothers (MMs) are HIV-positive, PMTCT-experienced women who counsel less-experienced peers for optimal PMTCT outcomes [11]. This paper presents baseline characteristics of MoMent participants and reports correlates of facility delivery in this study population.

## Methods

### Study design and setting

**Fig. 1:**
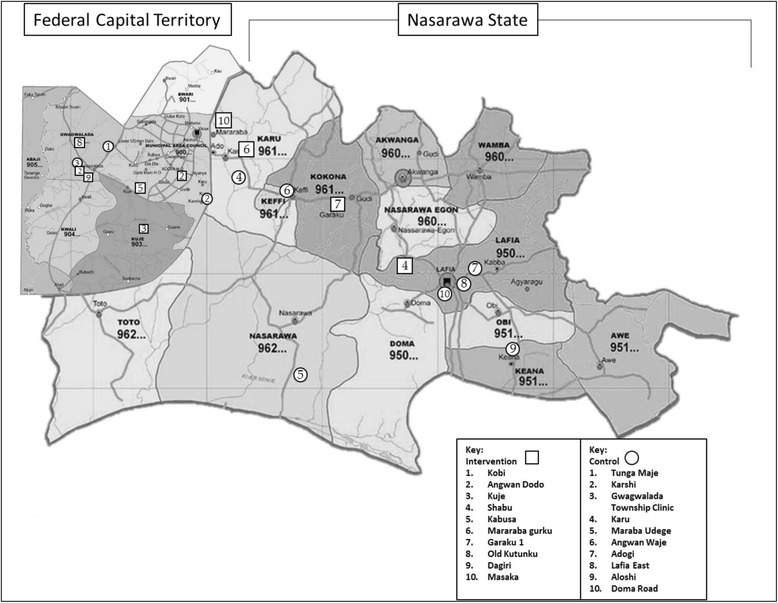
Map of Study States and Study Sites in North-Central Nigeria. Copyright 2017 by nigeriazipcodes.com (https://nigeriazipcodes.com/). Adapted with permission

The MoMent study was a prospective matched cohort study conducted in rural areas of the Federal Capital Territory and Nasarawa State (Fig. 1). The study states were located in North-Central Nigeria, which, at 5.8%, has the highest zonal HIV prevalence among pregnant women [12]. HIV prevalence among general populations in rural areas was also highest in the North-Central zone at 3.7% [12]. As at 2013, the two study states’ combined population comprised approximately 62% Christians and 34% Muslims [7, 13].

The MoMent study was implemented at public, state-owned and PEPFAR (United States’ President’s Emergency Plan for AIDS Relief)- supported primary healthcare centers (PHCs) located in 20 different towns and villages in 9 local government areas (districts) across the 2 study states (Fig. 1).

**Fig. 2:**
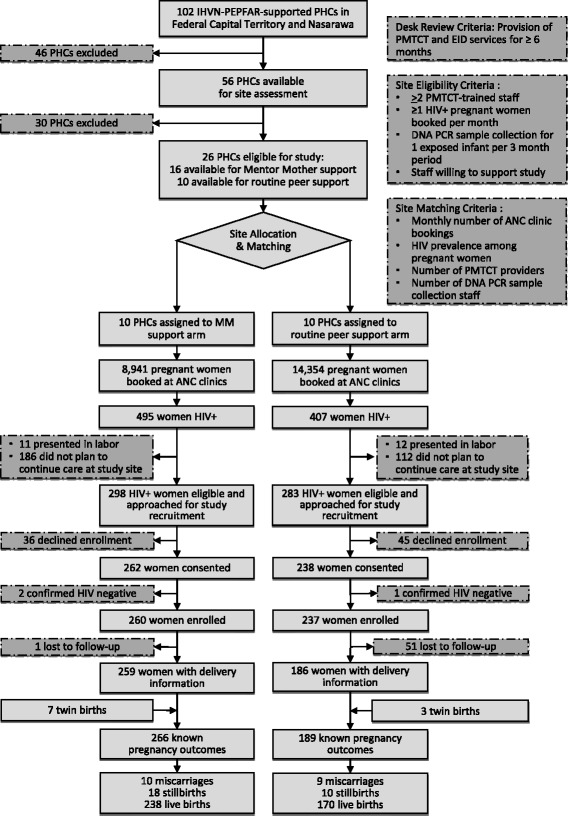
Site Selection and Participant Recruitment

The study intervention was structured peer support from trained, closely-supervised MMs; this was compared to loosely-organized, less-supervised routine peer support (PS). The structured MM intervention consisted of intensive baseline training, a detailed scope of work, close mentoring and supervision by a designated Mentor Mother supervisor, a standardized logbook for documentation of field activities, and periodic performance evaluations [10]. Due to PMTCT programmatic requirements for trained peer counselors at some eligible sites, the study could not be randomized. Ultimately, 10 of 16 intervention-eligible PHCs were matched with 10 routine PHCs based on 4 criteria: monthly number of new ANC client bookings, HIV prevalence among ANC clients, number of DNA PCR sample collection staff, and number of PMTCT staff (Fig. 2).

### Enrolment of study participants

We recruited two cohorts of HIV-positive pregnant women from ANC clinics, to receive either MM support or routine PS. Inclusion and exclusion criteria, recruitment and consent procedures and sample size estimations have already been described [10]. Briefly, sample size was 480 mother-infant pairs, and pregnant women ≥15 years old of any gestational age were recruited provided they had made at least 1 ANC visit prior to delivery. Women presenting for the first time in labor were not enrolled. Additionally, eligible participants had not previously received services from, or functioned as a peer counselor themselves. Both ART-naïve and ART-experienced women who planned to continue receiving services at the PHC enrollment site were recruited. HIV screening was performed per routine at ANC clinics of all study sites as follows: for each pregnant woman newly-booking whose HIV status was unknown or previously known to be HIV-negative, a rapid HIV test was performed. Group pre-test counseling was provided to eligible ANC clients by healthcare workers or peer counselors; individual client HIV testing was performed by healthcare workers only, then post-test counseling was individually provided to both HIV-negative and positive clients. HIV-positive clients were then counseled on ART, PMTCT, disclosure and male partner testing by healthcare workers and/or peer counselors, after which the HIV-positive clients were linked to a peer counselor at the intervention or control sites. All study participants received Option B PMTCT services per national guidelines [14] during recruitment and follow-up, including initiation of maternal ART regardless of CD4 count at booking.

### Baseline and delivery data collection

Participant baseline and delivery data were collected by face-to-face interview and abstraction from medical records using standardized forms (Additional files 1 and 2). Information collected were socio-demographic data including age, religion, residence address; clinical data including obstetric history, ART status and delivery information. The study PHCs were part of a primary healthcare system that lacked electronic medical records, or for the few that had one, had no cross-facility electronic communication or linkage of medical records. As such the majority of medical record documentation was manual and available only at the study site as far as any routine data (including delivery information) were concerned. Therefore, delivery data for women who did not deliver at the study facility were obtained directly from study participants and/or their peer counselors.

### Statistical analysis

Frequencies and distributions of participant baseline characteristics were computed, and Chi square test compared proportions between study arms. These characteristics were also compared between women who were lost to follow-up before delivery and those who remained in care to assess for difference between the two groups. The outcome of interest was place of delivery; specifically whether women delivered at a health facility or not. Place of delivery and correlates analyses were restricted to women with available delivery data. In order to assess correlates of facility delivery, multivariate logistic regression with generalized estimating equation was used to account for clustering. Covariates assessed included maternal baseline characteristics such as age, educational level, marital status, religious affiliation, and HIV/ART status at enrollment. For women who were lost to follow-up before delivery and therefore had no delivery information available, demographic and obstetric characteristics were used to impute places of delivery in a sensitivity analysis. The imputation model included the following variables: study arm (MM vs routine PS), place of delivery (facility vs non-facility) age, education, marital status, religion, distance lived to the clinic, gestational age at booking, gravidity (primigravid vs multigravid), HIV disclosure status and timing of HIV diagnosis (current vs previous pregnancy).

All associations were presented as adjusted odds ratios with 95% confidence intervals (CIs). Estimates whose CIs excluded 1 and comparisons with *p* < 0.05 were considered statistically significant. Statistical analyses were conducted using Statistical Analysis Software, version 9.3 (SAS Institute Inc., Cary, NC).

## Results

A total of 497 HIV-positive pregnant women were enrolled between April 2014 and September 2015, and were recruited from among 902 (3.9%) women testing positive out of 23,295 pregnant women (Fig. 2). Out of the 497 women enrolled, 263 (52.9%) were enrolled at first ANC visit, and 234 (47.1%) at subsequent ANC visits.

### Baseline characteristics of enrolled hiv-positive pregnant women

Baseline sociodemographic, obstetric and clinical data are presented in Table 1.

**Table 1:** Baseline characteristics of enrolled HIV-positive pregnant Women

Characteristic	Total (*N* = 497) n (%)	Mentor Mother Support (*N* = 260) n (%)	Routine Peer Support (*N* = 237) n (%)	p–value^a^
Age, years				
< 21	54 (10.9)	25 (9.6)	29 (12.2)	
21–30	352 (70.8)	185 (71.2)	167 (70.5)	
> 30	91 (18.3)	50 (19.2)	41 (17.3)	0.59
Formal Education				
< Secondary	252 (50.7)	110 (42.3)	142 (59.9)	
≥ Secondary	245 (49.3)	150 (57.7)	95 (40.1)	**<0.01**
Religion				
Muslim	178 (35.8)	58 (22.3)	120 (50.6)	
Christian	319 (64.2)	202 (77.7)	117 (49.4)	**<0.01**
Marital status				
Single^b^	25 (5.0)	15 (5.8)	10 (4.2)	
Married	470 (95.0)	244 (94.2)	226 (95.8)	0.43
Missing	2	1	1	
Employment status				
Unemployed	358 (72.0)	168 (64.6)	190 (80.2)	
Employed-unskilled	68 (13.7)	47 (18.1)	21 (8.8)	
Employed-skilled	71 (14.3)	45 (17.3)	26 (11.0)	**<0.05**
Gravidity				
Multigravid	402 (80.9)	202 (77.7)	200 (84.4)	
Primigravid	95 (19.1)	58 (22.3)	37 (15.6)	0.06
GA at booking, weeks				
0–12	99 (20.4)	45 (17.8)	54 (23.2)	
13–27	254 (52.2)	138 (54.5)	116 (49.8)	
28–42	133 (27.4)	70 (27.7)	63 (27.0)	0.32
Missing	11	7	4	
Disclosed HIV status^c^				
No	129 (26.0)	65 (25.0)	64 (27.0)	
Yes	368 (74.0)	195 (75.0)	173 (73.0)	0.61
Distance to facility, km				
< 5	270 (54.6)	152 (58.9)	118 (49.8)	
5–10	76 (15.3)	35 (13.6)	41 (17.3)	
> 10	149 (30.1	71 (27.5)	78 (32.9)	0.12
Missing	2	2	0	
Newly HIV-diagnosed^d^				
No	197 (39.7)	112 (43.1)	85 (36.0)	
Yes	299 (60.3)	148 (56.9)	151 (64.0)	0.11
Missing	1	0	1	
ART regimen^e^				
EFV-based	330 (66.4)	183 (70.4)	147 (62.0)	
NVP-based	156 (31.4)	71 (27.3)	85 (35.9)	
LPV/r-based	11 (2.2)	6 (2.3)	5 (2.1)	0.12

*GA* gestational age, *km* kilometers

Bolded *p* values denote statistically significant results at *p* < 0.05

^a^Comparisons between the two study arms

^b^Includes single, divorced, widowed, and separated women

^c^Disclosure to male partner or family member

^d^None of the newly-diagnosed women and all of the previously-diagnosed women were on ART at booking

^e^EFV: efavirenz; NVP: nevirapine; LPV/r: ritonavir-boosted lopinavir

Across the MM and routine PS arms, most respondents (71.2% and 70.5% respectively) were between age 21 and 30 years. About half (*n* = 245, 49.3%) of enrolled women had received secondary or higher-level education; with more MM than routine PS arm participants represented in this category (57.7% vs 40.1%, *p* < 0.01). Similarly, there were more Christian women in the MM than the routine PS arm (77.7% vs 49.4%, *p* < 0.01). Almost all women (*n* = 470, 95.0%) were married, and most (*n* = 402, 80.9%) were multigravida. Median gestational age at booking was 20 weeks [interquartile range (IQR) 16–28 weeks] with no difference between arms. In total, 60.3% (*n* = 299) of participants were newly HIV-diagnosed, and 74% (*n* = 368) of all enrolled women reported having disclosed HIV status to a male partner or family member. With regard to ART regimen, two-thirds of enrolled women (*n* = 330, 66.4%) were on efavirenz-based therapy per national guidelines [14]. There were no significant differences between arms for distance lived from facility, HIV diagnosis and disclosure status, and ART regimen. There was insufficient data to evaluate baseline CD4 as only 103 (20.7%) participants had an available test result.

### Place of delivery and skilled birth attendant at delivery

Delivery data was available for 445 (89.5%) of the 497 enrolled women. Median gestational age at delivery was 36 weeks (IQR 32–40 weeks). Nearly two-thirds (*n* = 276, 62.0%) of the 445 women delivered at a health facility, and 149 (33.5%) delivered at home. A total of 14 (3.2%) women delivered at a traditional birthing center with traditional birth attendants (TBAs). The remaining women delivered at religious centers (*n* = 2, 0.4%), and in a vehicle or maternity home (*n* = 4, 0.9%).

Table 2 presents delivery data analyzed by skilled or unskilled attendant at birth.

**Table 2:** Skilled vs unskilled attendant at delivery for HIV-positive women in rural Nigeria

Attendant at Delivery	All Deliveries^a^ (*N* = 445) n (%)	Mentor Mother Arm (*N* = 259) n (%)	Routine Peer Support Arm (*N* = 186) n (%)
Skilled Birth Attendant			
Doctor	17 (3.9)	14 (5.5)	3 (1.6)
Midwife	81 (18.5)	52 (20.3)	29 (15.8)
Nurse	160 (36.4)	87 (34.0)	73 (40.0)
CHW	50 (11.4)	32 (12.5)	18 (9.8)
Total	308 (70.2)	185 (72.3)	123 (67.2)
Unskilled Birth Attendant			
TBA	71 (16.2)	38 (14.8)	33 (18.0)
Family member	21 (4.8)	11 (4.3)	10 (5.5)
None/Self	37 (8.4)	21 (8.2)	16 (8.7)
Neighbor	2 (0.4)	1 (0.4)	1 (0.6)
Total	131 (29.8)	71 (27.7)	60 (32.8)
Missing data	6	3	3

*CHW* community healthcare worker, *TBA* Traditional Birth Attendant

^a^Women with available delivery information out of 497 enrolled women

Approximately 70% of women delivered with a skilled birth attendant (SBA). Nurses comprised the largest proportion of SBAs at participants’ deliveries (36.4%), followed by midwives (18.5%) and community healthcare workers (11.4%), who, in Nigeria, are trained, formal health workers comprising community health extension workers [15] and community health officers. At 3.9%, doctors comprised the lowest proportion of SBAs at deliveries among enrolled women. TBAs were the most utilized unskilled birth attendants: 16% of enrolled women delivered with TBAs, followed by no attendant/self-delivery (8.4%), family members (4.8%), and neighbors (0.4%).

Overall, of the 445 women with delivery data, 19 (4.3%) experienced miscarriage (pregnancy loss at <20 weeks gestation); 28 (6.3%) experienced stillbirth (pregnancy loss at ≥20 weeks gestation), and 398 (89.4%) had live births.

### Correlates of facility delivery

Of 445 women with available data, 276 (62.0%) delivered at a health facility (Table 3). Secondary or higher education (aOR = 1.9, CI 1.1–3.2) and Christian religious affiliation (aOR = 1.4, 95% CI 1.0–2.0) were associated with higher odds of facility delivery. Additionally, primigravidity (aOR = 0.5, 95% CI 0.3–0.9), and newly-diagnosed HIV status (aOR 0.6, 95% CI 0.4–0.9) were associated with decreased odds of facility delivery. There was no difference in facility delivery rates for women exposed to MM vs routine PS (61.2% and 62.6%, respectively, *p* = 0.7). Multivariate analysis also showed no difference in the odds of facility delivery between study arms (aOR = 0.8, 95% CI 0.5–1.2). None of the other evaluated factors (age, marital status, employment status, distance lived to facility, gestational age at booking and disclosure status) correlated with facility delivery.

**Table 3:** Correlates of facility delivery among HIV positive pregnant women in rural Nigeria

Characteristic	All	Facility Delivery (*n* = 276)		Bivariate Analysis		Multivariate Analysis	
	*N* = 445^a^	n	%^b^	OR	95% CI	aOR	95% CI
Type of peer support							
Routine Peer Support	186	114	61.2	1.0			
Mentor Mother	259	162	62.6	1.1	(0.7–1.8)	0.8	(0.5–1.2)
Age, years							
≤ 20	44	23	52.3	1.0		1.0	
21–30	317	199	62.8	1.1	(0.5–2.2)	1.4	(0.8–2.5)
≥ 31	84	54	64.3	1.1	(0.5–2.1)	1.2	(0.6–2.4)
Education							
< Secondary	217	111	51.1	1.0		1.0	
≥ Secondary	228	165	72.4	**2.4**	**(1.5–3.8)**	**1.9**	**(1.1–3.2)**
Marital status							
Single^c^	23	11	47.8	1.0			
Married	421	265	63.0	1.7	(0.5–5.1)	2.0	(0.7–6.0)
Employment status							
Unemployed	315	187	59.4	1.0		1.0	
Employed-unskilled	65	42	64.6	1.0	(0.6–1.7)	0.9	(0.5–1.6)
Employed-skilled	65	47	72.3	1.5	(0.8–2.9)	1.2	(0.6–2.6)
Religion							
Muslim	148	78	52.7	1.0		1.0	
Christian	297	198	66.7	**1.7**	**(1.3–2.4)**	**1.4**	**(1.0–2.0)**
Distance to facility							
< 5 km	240	153	63.8	1.0		1.0	
5–10 km	67	47	70.1	0.7	(0.3–1.7)	0.7	(0.3–1.7)
> 10 km	136	76	55.9	0.8	(0.5–1.2)	0.9	(0.5–1.5)
GA at booking, weeks							
0–12	85	48	56.5	1.0			
13–27	232	155	66.8	1.4	(0.8–2.3)	1.4	(0.7–2.8)
28–42	117	68	58.1	0.9	(0.5–1.6)	0.9.	(0.5–1.7)
Gravidity							
Multigravid	359	216	69.8	1.0		1.0	
Primigravid	86	60	60.2	**0.6**	**(0.3–1.0)**	**0.5**	**(0.3–0.9)**
Disclosed HIV status^d^							
No	115	68	59.1	1.0		1.0	
Yes	330	208	63.0	1.3	(0.8–1.9)	1.3	(0.8–0.9)
HIV-diagnosis status							
Previously-diagnosed	180	124	68.9	1.0		1.0	
Newly-diagnosed	264	152	57.6	**0.7**	**(0.4–1.0)**	**0.6**	**(0.4–0.9)**

*OR* Odds ratio, *aOR* adjusted odds ratio, *CI* Confidence Interval, *GA* gestational age

Bolded ORs, aORs and CIs denote statistically significant results

^a^Women with available delivery information out of 497 enrolled women

^b^Denotes row percentage of women with a facility delivery (n/N)*100

^c^Includes single, divorced, widowed and separated women

^d^Disclosure to male partner or family member

Due to the differential loss to follow-up of 0.4% (1/260) and 22% (51/237) among women in the MM and routine PS arms respectively, sensitivity analysis was performed. Sensitivity analysis in Table 4 demonstrates similar results to those in Table 3, with the exception of Christian affiliation, which was initially weakly correlated with facility delivery but in sensitivity analysis lost significance.

**Table 4:** Correlates of facility delivery among HIV positive pregnant women in Rural Nigeria: sensitivity analysis^a^

Characteristic	All	Facility Delivery (*n* = 289)		Bivariate Analysis		Multivariate Analysis	
	*N* = 497	n	%	OR	95% CI	aOR	95% CI
Education							
< Secondary	252	117	46.4	1.0		1.0	
≥ Secondary	245	172	70.2	**2.6**	**(1.7–3.9)**	**2.1**	**(1.3–3.3)**
Religion							
Muslim	178	87	48.9	1.0		1.0	
Christian	319	202	63.3	**1.7**	**(1.2–2.4)**	1.2	(0.8–1.9)
Gravidity							
Multigravid	402	226	56.2	1.0		1.0	
Primigravid	95	63	66.3	**0.6**	**(0.4–1.0)**	**0.6**	**(0.3–0.9)**
HIV-diagnosis status							
Previously-diagnosed	197	132	67.0	1.0		1.0	
Newly-diagnosed	299	157	52.5	**0.6**	**(0.4–0.9)**	**0.5**	**(0.3–0.8)**

*OR* Odds ratio, *aOR* adjusted odds ratio, *CI* Confidence Interval, *GA* gestational age

Bolded ORs, aORs and CIs denote statistically significant results

^a^Only statistically significant results are displayed

## Discussion

Our results show that neither type of peer support was associated with facility delivery. It was expected that facility delivery rates would be higher among women in the MM intervention arm. The non-correlation of peer support with facility delivery is likely due to a multiplicity of dominant factors. Some of these factors would not be amenable to significant modulation by peer support. Examples would be timing and urgency of labor and availability of 24/7 delivery services at the nearest accessible facility, transport to facility especially if onset of labor was at an inconvenient time, and available funds for facility delivery fees, which are substantially higher than for routine visits. Furthermore, given the median gestational age at booking and at delivery of 20 and 36 weeks respectively, the period of time between MM-client engagement and delivery was relatively short and may not have allowed for enough time for the MM interventional peer support to establish outcomes that would be significantly different from that of routine support. Detailed analyses of the impact of postpartum peer support on MoMent’s primary and key secondary outcomes have been published elsewhere [16, 17].

Our results also demonstrate that women in this rural cohort were relatively young (mostly <30 years), and most being newly-diagnosed and ART-naïve at the time of booking. The young age of these women and high total fertility rate of 6.2 in rural Nigeria [7] suggest a potentially high demand for future PMTCT services for this population. Studies from other African countries indicate young age, late gestational age and new HIV diagnosis at ANC booking as predictors of poor utilization of, and high loss-to-follow-up in PMTCT care [8, 18–20]. That most women in our study population were multigravida yet newly-diagnosed highlights the likelihood of missed PMTCT opportunities for previous pregnancies.

With regard to ART regimen, nevirapine-based therapy was prescribed for nearly a third of participants, a significant proportion given the de-emphasis of nevirapine and emphasis on efavirenz for PMTCT in the national guidelines [14].

About half (49.3%) of women in this cohort had at least secondary-level education, and significantly more women recruited in the Mentor Mother intervention arm had higher-level education. This could influence study outcomes, as higher level of maternal education has been consistently reported as a determinant of maternal health and PMTCT service uptake in Nigeria and in other African countries [8, 21]. There was an overrepresentation of self-identified Christians (78%) in the MM arm compared to the routine peer support arm (50%). This finding is in contrast to the reported distribution of Christians (62%) and Muslims (34%) across the two study states [7, 13]. Muslim women have been found at higher risk of poor service uptake due to limited male partner involvement, spousal consent requirements, and reluctance to be attended to by male healthcare providers [22, 23]. Qualitative data from MoMent’s study setting suggested that significant economic dependence on male partners may pose a barrier to access and uptake of PMTCT services among Muslim women [24]. We found a higher proportion of unemployed women in the routine PS arm compared to the MM arm, however employment status was not correlated with facility delivery. A review by Belemsaga et al. [8] reported that maternal employment hampered PMTCT service utilization and compliance among HIV-positive African women, presumably due to time constraints. The same paper [8] and another from Nigeria [25] have also reported that women lacking financial resources are less likely to use antenatal and delivery services. It may be that in spite of “employed” or “unemployed” status for some women in our study, inadequate earnings and continued economic dependence on male partners may have interacted to ultimately make no difference in facility delivery rates.

Not surprisingly, nearly all women in the study were married. A national reproductive survey reported that nearly 75% of 15 to 49 year old females in rural North-Central Nigeria were married [13]. Shehu et al. reported that married Nigerian women in rural Northern Nigeria were more likely to face barriers to health service utilization than single or urban women, largely due to requirements for husband’s consent to access care [26]. Power dynamics, associated repercussions of partner status disclosure and lack of spousal support are some of the challenges faced by married women in PMTCT [24, 27, 28]. Fortunately, there was a relatively high level (~75%) of disclosure reported among our study participants, compared to pooled rates reported for partner (64%) or any (67%) disclosure among African women [29]. This has positive implications, as partner disclosure has been reported to improve uptake and adherence to PMTCT services [30, 31]. However, the high proportion of married study participants indicates that male partner engagement and disclosure, ideally as collaborative efforts between HIV-positive women, peer counselors and healthcare workers, should continue to be an integral part of PMTCT programming in our study setting and in similar communities.

With regard to delivery, nearly two-thirds (62%) of participants had a facility delivery, which is considerably higher than the 22% facility deliveries reported among rural Nigerian populations [7]. This large difference is likely due to our study population being HIV-positive; HIV-positive women, especially those previously diagnosed and multigravida have experience with facility-based care and health information secondary to HIV treatment. This was demonstrated in Kenya, where rate of facility delivery among HIV-positive women (47%) was higher than among uninfected women (40%) [32]. Furthermore, all women in our HIV-positive study population were exposed to some form of peer support regardless of study arm. While there were no differences between study arms, peer support may have also contributed to the overall higher facility delivery rates in our HIV-positive cohort compared to the general Nigerian population. Studies in other African countries have reported widely different rates of facility deliveries (34% to 77%) among HIV-infected women, however these wide variations correlated with wide variations in the background rates of general population facility deliveries in the study settings [32–34].

Consistent with national data [7, 13] and studies among both HIV-infected and uninfected women in similar settings [8], uptake of facility delivery in our study was higher among women with higher levels of education, specifically twice as high among those with secondary-level or greater education. Women who have at least completed primary school are more likely to understand the risk of non-facility delivery including attendance by unskilled personnel with unsafe obstetric practices that have higher risks for undesirable birth outcomes [8]. Studies have attributed reported differences in health-seeking behavior among women with different levels of education to the consumption of health messages [8]. This suggests that there may be a gap in the design and dissemination of such information, especially for women with lower-level education.

Albeit a weak correlation, Muslim women were less likely to have a facility delivery than Christians, which is consistent with findings on health service uptake from Nigerian national surveys [7, 13]. The poor uptake of maternal health services among Muslim women across Africa has been attributed to socio-cultural and religious practices that limit their decision-making power and increase dependence on male partners and other family and community members [22, 24, 35–37]. This phenomenon needs to be contextually explored in Nigeria and other countries with the aim to develop community-acceptable strategies to increase health service utilization among Muslim women.

Notably, the odds of having a facility delivery did not differ between women who enrolled in early or late pregnancy. This finding is in contrast with previous studies that have reported an association between early gestational age at booking and use of facility-based delivery and postpartum services [8, 32]. Our study participants were largely multigravida (81%) and all HIV-positive. Women with prior experience with ANC/PMTCT services may initiate ANC late especially in rural settings like that of our study, where financial and cost barriers have been reported [25, 36, 38]. However, these women’s HIV status as well as their awareness of the importance of facility delivery based on previous experience may encourage them to seek facility delivery even with late bookings. Furthermore, some facilities insist on women being pre-registered during the antenatal period in order to be prioritized for seamless delivery services at the onset of labor and peripartum. Thus, HIV-positive women who may not place high value on routine antenatal care but desire facility-based delivery services often pre-register, albeit late, in order to receive priority delivery care. Distance from facility and HIV status disclosure, which have been associated with facility delivery and other facility service uptake [8, 30, 39, 40] were also not found to be correlates in our study. Results from qualitative studies in MoMent’s setting suggest that 24/7 accessible services, affordable cost of services, minimized commodity stock-outs, and friendlier healthcare worker attitudes could lead to increased utilization of facility-based services, including infant deliveries, among HIV-positive women [38].

Our study is limited by the non-randomized study design. However, as occurred in our study, the real-world nature of implementation research may necessitate major design adjustments due to environmental and/or programmatic changes. Ultimately, there was overrepresentation of Christian as well as better-educated women in the MM arm. Given the known positive effect of maternal education on health service uptake and historically low uptake among Muslim women, the unbalanced recruitment may affect interpretation of the prospective study’s primary outcomes. Data on pregnancy outcomes (miscarriage, stillbirth and live birth) were collected for all study participants but are not reported in this paper because the study was not designed to evaluate pregnancy outcomes and their correlates, whether by place of delivery or by study arm. Factors influencing these different pregnancy outcomes are likely multiple and co-dependent, and our study did not collect enough of the data needed eg individual, community, facility and healthcare provider level-for robust analysis and interpretation.

## Conclusions

Our study provides information not only for improving facility delivery uptake, but for increasing maternal health service utilization. First-time and newly-diagnosed HIV-positive mothers regardless of age, educational level or religion need to be targeted for education and supportive counseling to enable them navigate both pregnancy and living with HIV in the crucial antenatal and perinatal periods. Early and intensive counseling and peer support may be most important for this population of women. Ultimately, for maximal benefit, it is important that HIV-positive women deliver at facilities that provide 24/7 services and are equipped to handle a broad range of emergencies.

Unanswered research questions remain such as whether early first trimester engagement of peer support results in improved facility delivery rates and pregnancy outcomes among HIV-positive women, the role of religion in facility delivery in this population-especially in rural settings, and optimizing TBA roles in maternal healthcare service delivery without compromising quality.

## Additional files

Additional file 1: Enrollment Form for pregnant HIV-positive women (Case Report Form 1). Form captures baseline socio-demographic, obstetric, and other clinical data for all women enrolled in the MoMent study. (PDF 113 kb)

Additional file 2: Delivery form for pregnant HIV-positive women (Case Report Form 4). Form captures delivery information including place of delivery, attendant at delivery, and maternal/infant outcomes at delivery, for all women enrolled in the MoMent study. (PDF 64 kb)

## Abbreviations

ANC: Antenatal care
aOR: adjusted Odds Ratio
ART: Antiretroviral therapy
ARV: Antiretroviral drugs
CI: Confidence interval
MM: Mentor mother
PHC: Primary healthcare center
PMTCT: Prevention of mother-to-child transmission of HIV
PS: Peer support
SBA: Skilled birth attendant
TBA: Traditional birth attendant
