# Supplementary material for: Correlates of facility delivery for rural HIV-positive pregnant women enrolled in the MoMent Nigeria prospective cohort study
Source: BMC Pregnancy Childbirth. 2017 Jul 14;17:227. doi: 10.1186/s12884-017-1417-2 (PMC5512933; doi:10.1186/s12884-017-1417-2)
Supplement: Supplementary file 1 — Enrollment Form for pregnant HIV-positive women (Case Report Form 1). Form captures baseline socio-demographic, obstetric, and other clinical data for all women enrolled in the MoMent study. (PDF 113 kb) [file 12884_2017_1417_MOESM1_ESM.pdf]

IHVN-INSPIRE MoMent

Enrollment Form: FORM 01

Pregnant Mother Information

Source: Client, ANC, PMTCT HCT & ARV registers

Socio-demographic Data

1. Date of enrollment: d d m m m y y
2. ANC/Hospital No.
3. Study ID:
4. Date of birth: d d m m m y y
5. Age:_____
6. Religion: Christian ○ Islam ○ Other ○
7. Educational status: None ○ Primary ○ Secondary ○ Tertiary ○ Non-formal ○
8. Occupation: Unemployed ○ Employed-skill ○ Employed-unskilled ○
9. Mobile/phone: _______________
10. Address:
11. Town/village:
12. How long have you lived in this Town/village (yrs):
13. Marital status: *Single* ○ *Married* ○ *Divorced* ○ *Widow* ○ *Separated* ○
14. Gravida _____ No. of previous pregnancies including this one
15. Para _____ No. of previous births/deliveries

ANC/PMTCT History

Source: Client, ANC, PMTCT HCT & ARV registers

16. Date of first ANC visit: d d m m m y y
17. Last menstrual period: d d m m m y y
18. When was HIV diagnosed: Past pregnancy ○ Present pregnancy ○
19. Month/Year of HIV diagnosis: _____/ ____
20. No. of pregnancies post HIV diagnosis:
21. Have you disclosed your HIV status? Yes ○ No ○
22. Who did you disclose to: Partner/spouse ☐ Close friend ☐ Family member/relative ☐ Other
23. Gestational age at booking (weeks): ______
24. Expected date of delivery: d d m m m y y
25. Where do you plan to deliver your baby? This facility ☐ Home ☐ TBA ☐ Religious center ☐ Other
26. HAART Regimen: NVP-based ☐ EFV based ☐ Other (please specify): / /
27. Current WHO staging for this visit: *Stage I* ☐ *Stage II* ☐ *Stage III* ☐ *Stage IV* ☐ *Immunological CD4* ☐

28a. Most recent hemoglobin/PCV:
28b. Date: d d m m m y y

29a. Baseline CD4: *(cells/mm3)*
29b. Date: d d m m m y y

30a. Most recent viral load test: *(copies/ml)*
30b. Date: d d m m m y y

31. Counseled on infant feeding methods? *Yes* ☐ *No* ☐

31. Infant feeding choice made by mother: *EBF* ☐ *EBMS* ☐ *MF* ☐ *Other*

**MoMent staff Name:**	**Designation:**	**Date:**	**Sign:**

This form to be filled by Research Associate or Site Research Officer
