# Supplementary material for: Correlates of facility delivery for rural HIV-positive pregnant women enrolled in the MoMent Nigeria prospective cohort study
Source: BMC Pregnancy Childbirth. 2017 Jul 14;17:227. doi: 10.1186/s12884-017-1417-2 (PMC5512933; doi:10.1186/s12884-017-1417-2)
Supplement: Supplementary file 2 — Delivery form for pregnant HIV-positive women (Case Report Form 4). Form captures delivery information including place of delivery, attendant at delivery, and maternal/infant outcomes at delivery, for all women enrolled in the MoMent study. (PDF 64 kb) [file 12884_2017_1417_MOESM2_ESM.pdf]

IHVN-INSPIRE MoMent

Delivery Report: FORM 04

Source: Delivery, PMTCT-ARV registers, pharmacy records

Delivery Information

1. Date of delivery: d d m m m y y
2. Time of delivery: H M
3. ANC No.

5. Mother's ID:
6. Child's ID:
7. Child's sex: Male ☐ Female ☐

8. Site of delivery: ANC Health facility ☐ Birthing center (*e.g. TBA*) ☐ Home ☐ Religious center ☐ Other
Other health facility

9. Delivery attended by: Midwife ☐ Nurse ☐ TBA ☐ None ☐ Other

10. Mode of delivery: Spontaneous vaginal delivery ☐ Caesarian section ☐ Instrument delivery (forceps/vacuum) ☐

11. Infant delivery outcome: Alive ☐ Dead ☐
12. Infant birth weight: kg
13a. Maternal ART: NVP-based ☐ EFV-based ☐
14. Maternal outcome: Alive ☐ Dead ☐
13b. Other ART: / /

14. Infant NVP given: Yes ☐ No ☐
15. First infant NVP given at *(time)*: H M

16. First infant NVP given on *(date)*: d d m m m y y

MoMent staff Name:	Designation:	Date:	Sign:

This form to be filled by Research Associate or Site Research Officer
